# Advancing Parkinson’s disease detection through multi-dimensional machine learning: a comprehensive framework using wearable movement sensor analytics

**DOI:** 10.3389/fphys.2025.1737585

**Published:** 2026-01-05

**Authors:** Jun-Zhi Xiang, Qin-Yong Wang, Zhi-Bin Fang, James A. Esquivel, Xue-Yan Li, Xiao-Qun Xu

**Affiliations:** 1 Emergency Department, The First Affiliated Hospital of Wenzhou Medical University, Wenzhou, Zhejiang, China; 2 School of Artificial Intelligence, Zhejiang College of Security Technology, Wenzhou, Zhejiang, China; 3 School of Electronic and Electrical Engineering, Wenzhou University of Technology, Wenzhou, Zhejiang, China; 4 School of Chemistry, Dalian University of Technology, Dalian, Liaoning, China; 5 R&D Department, Chongqing Rongguan Technology Co., Ltd., Chongqing, China; 6 School of Software Technology, Zhejiang University, Ningbo, Zhejiang, China; 7 Graduate School, Angeles University Foundation, Angeles, Philippines

**Keywords:** feature extraction, machine learning, Parkinson’s disease detection, SHAP analysis, wearable movement sensors

## Abstract

**Background:**

wearable movement sensor technology shows promise for objective assessment of Parkinson’s disease (PD) motor symptoms, but optimal machine learning approaches and feature sets for accurate PD detection remain unclear. This study provides a comprehensive evaluation of classification algorithms, feature contributions, and optimization techniques for PD detection using wearable movement sensor data.

**Methods:**

We compared twelve diverse machine learning classifiers on motion sensor data, conducted systematic feature ablation studies across statistical, frequency-domain, dynamic, and complexity feature categories, optimized Random Forest parameters using three meta-heuristic algorithms, which is Particle Swarm Optimization (PSO), Improved Satin Swarm Algorithm (ISSA), and Enhanced Whale Optimization Algorithm (EWOA), and performed SHAP value analysis to identify the most influential features and their impact patterns.

**Results:**

Random Forest demonstrated superior performance (86.7% accuracy) among all classifiers. Statistical features contributed most significantly to classification performance, while complexity, dynamic, and frequency domain features provided complementary information. PSO-optimized Random Forest achieved 87.65% accuracy, outperforming other optimization approaches. SHAP analysis identified entropy-based measures and standard deviations as the most influential features, with accelerometer-derived complexity measures driving high-probability PD predictions and gyroscope-derived measurements dominating low-probability outcomes.

**Conclusion:**

Ensemble-based methods effectively capture the complex, non-linear relationship between movement characteristics and PD diagnosis. Comprehensive feature extraction frameworks incorporating multiple movement dimensions significantly enhance detection accuracy. The asymmetric feature influence patterns for positive versus negative predictions align with clinical understanding of PD as a disorder characterized by altered movement complexity and variability. These findings provide a foundation for developing accurate, interpretable wearable monitoring systems for Parkinson’s disease detection and management.

## 1 Introduction

Parkinson’s disease is a chronic, progressive neurodegenerative disorder that significantly affects motor and non-motor functions. Motor symptoms, such as tremors, rigidity, and bradykinesia, are the hallmark of the disease, while non-motor symptoms, including cognitive impairments, autonomic dysfunction, and sleep disturbances, further reduce the quality of life. Research indicates that the global prevalence of Parkinson’s disease is 94 per 100,000 people, with a significant increase in incidence with age (Khani et al., 2024). Early and accurate diagnosis is critical for effective intervention and management, yet it remains challenging due to the disease’s heterogeneous progression and the subtlety of early-stage symptoms (Siderowf et al., 2023).

Traditional diagnostic approaches for PD rely on clinical observations, patient-reported symptoms, and rating scales such as the Unified Parkinson’s Disease Rating Scale (UPDRS) (Guerra et al., 2023). While these tools provide valuable insights, they are inherently subjective, prone to inter-rater variability, and dependent on observable symptoms that often emerge in the later stages of the disease. As a result, there is an urgent need for objective, data-driven diagnostic methods that can identify PD at earlier stages (Mei et al., 2021).

In recent years, wearable devices such as smartwatches have emerged as promising tools for PD monitoring and diagnosis (Sotirakis et al., 2023). Equipped with accelerometers, gyroscopes, and other motion sensors, these devices capture high-resolution time-series data reflecting an individual’s movements. By analyzing this data, it is possible to detect subtle motor impairments and other movement-related abnormalities indicative of PD. The use of wearable devices offers several advantages, including non-invasiveness, scalability, and the ability to monitor individuals in real-world environments over extended periods (Powers et al., 2021).

This study introduces a novel framework for PD detection using wearable movement sensor data, emphasizing activity-robust feature extraction, class imbalance handling, and explainable modeling.

The key contributions of this research are as follows:

1. Multi-dimensional Feature Extraction Framework: We propose a comprehensive feature extraction strategy that systematically integrates statistical, frequency-domain, dynamic, and complexity-based characteristics from smartwatch sensor data. This unified framework uses activity-agnostic features that do not depend on task-specific biomechanical models, thereby offering potential robustness across diverse movement contexts.
2. PSO-optimized Classification Architecture: We develop a novel classification approach that leverages Particle Swarm Optimization for parameter tuning, automatically identifying optimal hyperparameter configurations. This optimization strategy significantly improves the model’s discriminative power in distinguishing between PD patients and healthy controls.
3. Interpretable Feature Analysis Framework: We incorporate SHapley Additive exPlanations (SHAP) analysis to provide transparent insights into feature importance and model decision-making processes. This interpretability mechanism helps identify the most significant movement characteristics contributing to PD detection, enhancing the clinical relevance and trustworthiness of our approach.
4. Systematic Performance Validation: We conduct comprehensive experimental evaluations, examining model performance through multiple metrics including accuracy, precision, recall, and F1-score. The evaluation framework provides robust validation of the proposed method’s effectiveness in real-world PD detection scenarios.

This study addresses critical gaps in PD detection research by proposing a method that generalizes across activities and offers interpretable results. The ability to identify activity-robust features ensures robustness in uncontrolled, real-world settings, making the proposed framework suitable for scalable deployment in wearable-based health monitoring systems. Furthermore, the integration of SHAP analysis aligns with the increasing emphasis on explainable artificial intelligence (XAI) in healthcare, providing clinicians with actionable insights into the factors driving model decisions.

Through this study, we aim to contribute a robust, interpretable, and activity-robust framework for PD detection, advancing the capabilities of wearable-based diagnostic systems in clinical and real-world applications.

## 2 Related work

Parkinson’s disease is typically diagnosed through clinical evaluations involving neurological examinations and patient history assessments. Tools like the UPDRS provide standardized metrics to assess disease severity but are inherently subjective and depend on the clinician’s expertise (Nair et al., 2021; Ramdhani et al., 2018). Additionally, overlapping symptoms with other neurodegenerative disorders complicate early diagnosis.

To overcome these challenges, researchers have explored data-driven approaches for automated PD detection (Ammous et al., 2024; Dhivyaa et al., 2024). Machine learning algorithms have leveraged diverse data types, such as voice recordings, handwriting samples, and motion sensor data, to identify PD-related patterns (Islam et al., 2024; Kamran et al., 2021; Veetil et al., 2024). Studies using voice data have extracted features like jitter, shimmer, and harmonics-to-noise ratio to differentiate PD patients from healthy individuals with promising accuracy (Akbarzadeh-T et al., 2021). Similarly, handwriting analysis has investigated features such as tremor frequency and pressure variations to detect motor impairments (Ngo et al., 2024; Palsapure et al., 2024; Goel et al., 2020). While these approaches show promise, they often require specific tasks for data collection, limiting their generalizability to real-world applications.

Wearable devices equipped with sensors such as accelerometers, gyroscopes, and heart rate monitors have gained prominence in healthcare for enabling continuous, non-invasive monitoring of physiological and behavioral data. These devices offer significant potential for early disease detection and management (Peng et al., 2023).

In the context of PD, wearable movement sensors have been utilized to analyze motor symptoms by assessing gait patterns, tremor frequencies, and activity levels. For instance, These features were useful for distinguishing between subtypes and monitoring disease progression. The findings suggest that wearable movement sensors could aid early diagnosis and personalized treatment by identifying subtype-specific gait biomarkers (Zhang et al., 2024; Rovini et al., 2017). However, these methods often rely on task-specific models that require participants to perform predefined activities, such as walking or writing, reducing their applicability in unconstrained environments where individuals engage in diverse activities.

A critical limitation of wearable-based PD detection is the dependence on task-specific features, such as stride length for walking or tremor amplitude during handwriting. While effective in controlled environments, these features lack generalizability to real-world scenarios.

Recent studies have explored activity-robust feature extraction for detecting PD using wearable movement sensors. A method was developed that combines multilevel features from spectral, temporal, and sensor domain data to assess motor fluctuations in PD patients (Behnaz et al., 2019). The impact of sensor types, sampling rates, and feature sets on PD symptom detection accuracy was investigated, with findings suggesting that simplified measurement characteristics could maintain performance while reducing computational burden (Shawen et al., 2020). Additionally, it was demonstrated that gyroscope data slightly improved bradykinesia detection, while tremor detection accuracy decreased with lower sampling rates (Shawen et al., 2020). An optimized PD detection method using dynamic kinematic features extracted from specific phases of handwriting tasks was proposed, achieving high accuracy through machine learning techniques (Shin et al., 2024). These studies highlight the potential of activity-robust features and optimized data collection strategies for robust PD detection using wearable movement sensors.

XAI techniques are crucial for enhancing transparency and trust in healthcare machine learning models. SHAP and LIME are two prominent model-agnostic methods that provide insights into model predictions (Arjunan, 2021; Inukonda and Rajasekhara Reddy Tetala, 2024). These techniques help bridge the gap between technical outputs and clinical applications, addressing the “black-box” problem in AI (Inukonda and Rajasekhara Reddy Tetala, 2024). XAI methods are particularly important in high-stakes medical fields like diagnostics and treatment personalization, where interpretability is essential for ethical decision-making and regulatory compliance (Arjunan, 2021). Studies have demonstrated the effectiveness of SHAP and LIME in various healthcare applications, including melanoma prediction and diabetic retinopathy diagnosis (Shobeiri, 2024). By providing explanations for model decisions, XAI techniques enable clinicians to understand, trust, and safely apply AI recommendations, ultimately improving clinical workflows and patient outcomes (Arjunan, 2021; Inukonda and Rajasekhara Reddy Tetala, 2024; Shobeiri, 2024).

## 3 Methodology

In this study, a comprehensive methodology is proposed to detect Parkinson’s Disease from smartwatch sensor data, utilizing time-series accelerometer and gyroscope readings. The methodology consists of five primary steps: (1) data collection and preprocessing, (2) feature extraction, (3) class balancing, and (4) experimental design. These steps ensure that the model is both accurate and robust for detecting PD-related motion patterns.

### 3.1 Dataset description

The Parkinson’s Disease Smartwatch Dataset is a publicly available dataset from PhysioNet that contains motion sensor recordings collected using a smartwatch worn by participants with and without Parkinson’s disease (Varghese et al., 2024). It was collected from 2018 to 2021 at the University Hospital Münster, Germany. The data collection involved 469 participants, generating a total of 5,159 measurement steps. The data acquisition system consisted of two Apple Watch Series 4 smartwatches worn on both wrists and a smartphone running a custom application. During the data collection process, participants performed 11 different standardized movement tasks, each lasting between 10 and 20 s. The smartwatches simultaneously recorded acceleration and rotation signals throughout these tasks, which were specifically designed to provoke subtle movement pathologies.

The dataset includes both sensor measurements and participant information (Table 1). The sensor data comprises synchronized acceleration and rotation signals from both smartwatches during task execution. For privacy protection, all participants were assigned random unique identifiers, and temporal data was normalized to start from zero.

**TABLE 1:** Participant demographic and clinical characteristics.

Characteristic	Healthy controls	Parkinson’s disease
Age, years (mean ± SD)	62.9 ± 12.5	65.4 ± 9.6
Age range, years	40–90	42–85
BMI, kg/m^2^ (mean ± SD)	28.8 ± 19.6	27.0 ± 5.0

This comprehensive dataset provides a robust foundation for developing and validating machine learning models aimed at detecting and analyzing movement disorders through digital biomarkers.

### 3.2 Data preprocessing

The data preprocessing stage consisted of two main components: data cleaning and alignment, followed by label filtering. In the first component, raw sensor data underwent cleaning procedures to remove noise and artifacts. The subsequent label filtering process involved carefully selecting and validating the movement task labels, ensuring only correctly labeled and complete movement sequences were retained for the analysis.

#### 3.2.1 Data cleaning and alignment

In the initial preprocessing step, the raw sensor data from different subjects is aligned to ensure that all time-series sequences have the same length. Sequences were aligned to the length of the longest recording within each cross-validation fold by zero-padding shorter sequences at the end or truncating the terminal portion of longer ones. All preprocessing was performed strictly after train/validation/test splitting to prevent data leakage. Zero-padding or truncation was chosen because it is the standard approach for this publicly available dataset, introduces only neutral values, and avoids the artificial dynamics that interpolation or reflection padding can induce in tremor- and bradykinesia-sensitive signals.

This ensures a consistent input format suitable for machine learning models. Additionally, labels from a separate metadata file are mapped to the corresponding time-series data.

Given the nature of wearable movement sensors, noise and missing values can distort the time-series signals. Therefore, forward-filling is used to impute missing values, preserving the continuity of the signal, especially when data is sparse or corrupted. After padding/truncation, sequences were segmented into overlapping 5-s windows to preserve temporal dynamics and mitigate artifacts.

#### 3.2.2 Label filtering

Since the dataset includes multiple classes of conditions, the analysis is focused on distinguishing between healthy individuals and those diagnosed with Parkinson’s Disease. Therefore, labels are filtered to only include “Healthy” and “Parkinson’s” conditions, and the corresponding time-series samples are retained.

### 3.3 Feature extraction

A key design principle of the proposed framework is the use of activity-agnostic features. Unlike many prior studies that extract gait-specific, drawing-specific, or tapping-specific parameters, all features employed here can be computed on any 5-s accelerometer or gyroscope segment irrespective of the underlying motor task. This deliberate choice aims to reduce task dependency at the feature level.

Feature extraction was performed on overlapping 5-s windows extracted from the entire duration of each task recording. This window length was selected because it captures multiple cycles of physiological tremor (four to eight Hz) while remaining computationally efficient.

A 50% overlap (2.5-s stride) was used to preserve temporal continuity and avoid boundary effects when computing frequency-domain features, which is standard for physiological time-series analysis.

Statistical, frequency-domain, dynamic, and complexity features were combined because PD motor impairments manifest across multiple temporal scales (steady-state, oscillatory, and transient), and ensemble feature sets consistently outperform single-domain approaches in biomedical classification tasks.

#### 3.3.1 Statistical features

Statistical features provide insights into the central tendency and dispersion of the data (Jalal et al., 2020). For both accelerometer and gyroscope signals, the following statistical metrics are computed:

Mean ( $\mu_{x}$ ) and Standard Deviation ( $\sigma_{x}$ ) (Equation 1):

$$\mu_{x}=\frac{1}{N}\sum_{i=1}^{N}x_{i},\;\sigma_{x}=\sqrt{\frac{1}{N}\sum_{i=1}^{N}{\left(x_{i}-\mu_{x}\right)}^{2}}$$ (1)

where $x_{i}$ represents the sensor value at time i , $\mu_{x}$ is the mean, and $\sigma_{x}$ is the standard deviation. These features are computed for each axis of both the accelerometer and gyroscope signals.

Maximum (max(x)) and Minimum (min(x)): These values capture the extremities of the signal range.

#### 3.3.2 Frequency-domain features

Frequency-domain analysis is essential for identifying oscillatory patterns and periodic signals inherent in the motion data (Dong et al., 2020). The Power Spectral Density (PSD) is estimated for each sensor signal using the Welch method, which divides the signal into overlapping segments and computes the average power of the frequency components (Equation 2):

$$P\left(f\right)=\frac{1}{N}\sum_{i=1}^{N}{\left|X\left(f\right)\right|}^{2}$$ (2)

where $P\left(f\right)$ is the power at frequency f , and $X\left(f\right)$ is the Fourier transform of the signal segment.

From the PSD, we derive the Spectral Entropy, which measures the complexity and randomness of the signal in the frequency domain. Spectral entropy is calculated as Equation 3.

$$H_{\text{spec}}=-\sum_{f}P\left(f\right)\log_{2}P\left(f\right)$$ (3)

This metric is particularly useful for detecting irregularities in the movement patterns associated with Parkinson’s Disease.

#### 3.3.3 Dynamic features

Dynamic features capture the temporal changes in sensor signals, highlighting the rate of motion or variability over time (Nakano and Chakraborty, 2023). We compute the Rate of Change for each sensor signal as follows Equation 4:

$$r_{X}=\frac{1}{N-1}\sum_{i=1}^{N-1}\left|\frac{x_{i+1}-x_{i}}{x_{i}}\right|$$ (4)

where $r_{X}$ represents the rate of change in the sensor signal, and $x_{i}$ denotes the value at time step i . This feature is computed for each axis of both the accelerometer and gyroscope signals.

#### 3.3.4 Complexity features

To capture the inherent complexity and unpredictability of motion, Shannon Entropy is computed for each sensor signal (Peng et al., 2014). The entropy quantifies the uncertainty or randomness of the signal’s distribution (Equation 5):

$$H_{\text{shannon}}=-\sum_{i=1}^{n}p\left(x_{i}\right)\log_{2}p\left(x_{i}\right)$$ (5)

where $p\left(x_{i}\right)$ is the probability distribution of the signal values, and n is the number of bins in the histogram. Higher entropy values generally correspond to more chaotic or irregular movements, which may be indicative of PD-related motor symptoms.

The extracted features are concatenated into a unified feature vector, representing a comprehensive profile of the sensor data for each time-series sample.

### 3.4 Class balancing

Due to class imbalance at the window level, the Synthetic Minority Oversampling Technique (SMOTE) was applied exclusively to the training set of each cross-validation fold after splitting. This produced an approximately 1:1 balanced ratio in training data only, while validation and test sets retained the original distribution. SMOTE was selected over random oversampling because it generates synthetic minority samples through nearest-neighbor interpolation, preserving the local structure of PD movement patterns in feature space. SMOTE works by generating synthetic instances through interpolation of existing minority class samples (Elreedy et al., 2023).

Mathematically, for a given minority class sample, SMOTE generates new synthetic samples as Equation 6:

$$x_{\text{new}}=x_{i}+\lambda \left(x_{n}-x_{i}\right)$$ (6)

where $x_{i}$ is a sample from the minority class, $x_{n}$ is a randomly selected neighbor of $x_{i}$ , and λ is a random scalar factor. This helps prevent overfitting and improves the generalization capability of the model.

### 3.5 Experimental design

To establish an optimal framework for Parkinson’s disease detection using wearable movement sensor data, we designed an experimental pipeline that integrates an advanced feature extraction method with PSO. In addition to evaluating multiple baseline models, we specifically emphasize the role of PSO in feature selection. Our approach leverages PSO to refine statistical, frequency-domain, dynamic, and complexity-based features, ensuring the most discriminative characteristics are selected for classification. By optimizing feature subsets, PSO enhances model interpretability, leading to improved classification performance. A comparative analysis between the baseline models and our proposed PSO-based feature selection approach provides insight into its effectiveness in enhancing classification outcomes.

#### 3.5.1 Baseline selection

We evaluated twelve widely adopted supervised learning algorithms spanning different algorithmic families:

Support Vector Machine (SVM): A margin-based discriminative classifier that finds an optimal hyperplane to separate different classes.

XGBoost: A gradient boosting framework that optimizes decision trees using an efficient boosting strategy.

LightGBM: A gradient boosting framework optimized for speed and performance.

K-Nearest Neighbors (KNN): An instance-based learning approach that classifies samples based on the majority class of their nearest neighbors.

Logistic Regression: A statistical model that uses a logistic function to model binary dependent variables.

Decision Tree: A tree-based classification method that partitions the data space into hierarchical regions.

Naive Bayes: A probabilistic classifier based on Bayes’ theorem with an assumption of independence among predictors.

Gradient Boosting Machine (GBM): An iterative boosting method that combines weak learners to create a strong predictive model.

Extreme Learning Machine (ELM): A neural network-based approach that randomly assigns weights and biases to hidden neurons and solves output weights analytically.

AdaBoost (Adaptive Boosting): An ensemble learning method that iteratively adjusts the weights of weak classifiers to enhance predictive accuracy.

Bagging: Bootstrap aggregating of base classifiers.

Random Forest: An ensemble of decision trees with feature bagging.

#### 3.5.2 Model architecture

**FIGURE 1:**
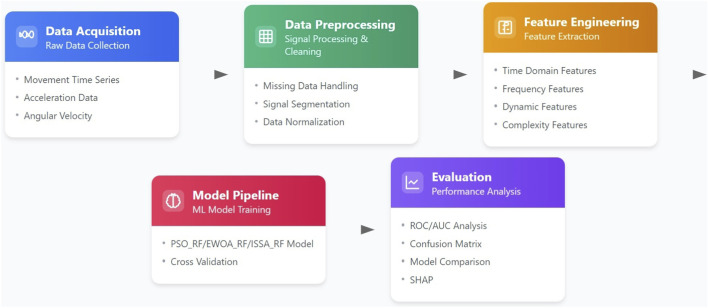
Model architecture.

The proposed Parkinson’s disease classification pipeline consists of multiple stages, starting from raw data acquisition to feature extraction, model training, and evaluation. The pipeline integrates conventional machine learning techniques with advanced optimization algorithms. Specifically, it employs PSO, ISSA, and EWOA to fine-tune the hyperparameters of a Random Forest (RF) classifier. The architecture is illustrated in Figure 1.

PSO is a population-based optimization algorithm inspired by the social behavior of bird flocks and fish schools. The algorithm maintains a swarm of particles, each representing a candidate solution, which moves through the search space based on position and velocity updates. The update rules for each particle are given as Equations 7, 8:

$$v_{i}^{\left(t+1\right)}=wv_{i}^{\left(t\right)}+c_{1}r_{1}\left(p_{i}^{\text{best}}-x_{i}^{\left(t\right)}\right)+c_{2}r_{2}\left(g^{\text{best}}-x_{i}^{\left(t\right)}\right)$$ (7)

$$x_{i}^{\left(t+1\right)}=x_{i}^{\left(t\right)}+v_{i}^{\left(t+1\right)}$$ (8)

where:

$x_{i}^{\left(t\right)}$ is the position of the i -th particle at iteration t .

$v_{i}^{\left(t\right)}$ is its velocity. 

$p_{i}^{\text{best}}$ represents the best position found by particle i so far. 

$g^{\text{best}}$ is the global best position found by any particle. 

w is the inertia weight controlling the influence of past velocities. 

$c_{1}$ , $c_{2}$ are acceleration coefficients for personal and global best attraction. 

$r_{1}$ , $r_{2}$ are random numbers sampled from [0, 1].

The ISSA is an enhanced version of the traditional Satin Swarm Algorithm, incorporating adaptive inertia weight and mutation strategies to avoid premature convergence. The position update in ISSA follows (Equation 9):

$$x_{i}^{\left(t+1\right)}=x_{i}^{\left(t\right)}+\alpha \cdot \text{rand}\cdot \left(x_{\text{best}}-x_{i}^{\left(t\right)}\right)$$ (9)

where:

$x_{i}^{\left(t\right)}$ is the position of the i -th individual.

α is a control parameter.

rand is a random number between [0, 1].

$x_{\text{best}}$ is the best position found so far.

EWOA improves the standard Whale Optimization Algorithm by incorporating chaotic mapping and nonlinear control parameter adjustments. The position update rule is given by Equation 10:

$$x_{i}^{\left(t+1\right)}=x_{i}^{\left(t\right)}+D\cdot e^{\text{bL}}\cdot \cos\left(2\pi L\right)$$ (10)

where:

D is the distance between the search agent and the best solution.

b is a constant controlling the logarithmic spiral shape. 

L is a random number in [-1, 1].

#### 3.5.3 Model algorithm

The proposed integrated framework for Parkinson’s Disease detection utilizing smartwatch sensor data is presented in Algorithm 1. The framework encompasses four primary stages: data preparation, label processing, feature extraction, and model optimization. In the data preparation phase, the algorithm processes raw time series data from smartwatch sensors, organizing it into a structured format while preserving subject identification and action type information. The label processing stage establishes a mapping between subjects and their medical conditions, specifically distinguishing between healthy controls (y = 0) and PD patients (y = 1). The feature extraction module implements a comprehensive set of feature calculations, including statistical metrics, frequency domain characteristics, dynamic movement patterns, and complexity measures, as defined by Equations 1–5. The final stage introduces a PSO-optimized classification approach, where particle swarm optimization dynamically adjusts the model parameters through velocity and position updates governed by Equations 7, 8. This optimization process iteratively refines the classification parameters to maximize diagnostic accuracy. The algorithm incorporates data normalization and SMOTE-based class balancing to ensure robust model performance, culminating in a comprehensive evaluation using multiple performance metrics.

**Input:**Raw time series data folder D_raw; Patient labels folder D_label
**Output:**Model performance metrics and ablation study results
1: [DATA PREPARATION]:
2: **Initialize empty lists time series list, metadata list**
3: **for**each file f in D_raw **do**
4:  Read sensor data matrix $x\in R\in \left(T\times F\right)$ ,where T is time steps and F is features
5:  Extract subject_id and action_type from filename
6:  Append X to time_series_list
7:  Append metadata to metadata_list
8: **end for**
9: [LABEL PROCESSING]:
10: Initialize empty dictionary labels_dict
11: **for**each file f in D_label **do**
12:   Read JSON data
13:   Extract subject_id and condition y_i ∈ {0,1}
14:   Add to labels_dict
15: **end for**
16: Merge labels with metadata
17: Filter data to include only Healthy (y = 0) and Parkinson’s (y = 1) cases
18: [FEATURE EXTRACTION]:
19: function ExtractFeatures(X):
20:  Split x into x_acc∈R^T×3 and
21:  if include_stats then
22:   Calculate statistical features (mean, std, max, min) using Equation 1
23:  end if
24:  if include_freq then
25:   Calculate frequency domain features using Equations 2, 3
26:  end if
27:  if include_dynamic then
28:   Calculate dynamic features (rate of change) using Equation 4
29:  end if
30:  if include_complexity then
31:   Calculate complexity features (entropy) using Equation 5
32:  end if
33:  Return concatenated feature vector
34: end function
35: [MODEL TRAINING AND EVALUATION]:
36: Initialize feature matrix $F\in R\;\left(N\times d\right)$ and label vector
37: **for**i = 1 to N **do**
38:  F [i] = ExtractFeatures (X_i)
39:  y [i] = label_i
40: **end for**
41: Normalize F: F_norm = (F - μ)/σ

Algorithm 1 Parkinson’s Disease Detection from Smartwatch Data.

42: Apply SMOTE: (F_balanced, y_balanced) = SMOTE(F_norm, y) (Equation 6)
43: [PSO OPTIMIZATION]:
44: Initialize particle swarm P with random positions and velocities
45: Set personal best position pbest and global best position gbest
46: **while not**reaching maximum iterations **do**
47:  **for**each particle p in P **do**
48:   Update velocity v using Equation 7
49:   Update position x using Equation 8
50:   Evaluate fitness using classification accuracy
51:   Update pbest and gbest if better solution found
52:  **end for**
53: **end while**
54: Train classifier θ with optimal parameters from PSO
55: Calculate metrics: accuracy, Precision, Recall, F1-score

## 4 Results

### 4.1 Baseline model comparison

To establish a comprehensive benchmark for Parkinson’s disease detection using wearable movement sensor data, we evaluated twelve diverse machine learning classifiers spanning multiple algorithmic families.

**FIGURE 2:**
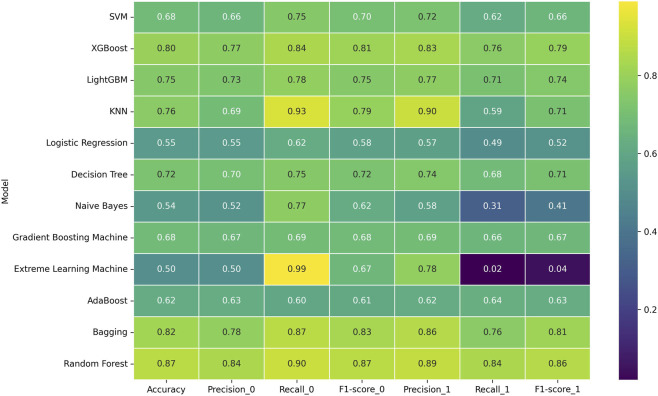
Model performance heatmap.

As illustrated in Figure 2, the Random Forest classifier demonstrated superior performance across all evaluation metrics, achieving 86.7% accuracy, 84% precision and 90% recall for the healthy class (class 0), and 89% precision and 84% recall for the Parkinson’s disease class (class 1). This balanced performance across both classes is particularly valuable in clinical diagnostic applications where both false positives and false negatives carry significant implications.

To provide a more rigorous assessment of model stability, we computed 95% bootstrap confidence intervals (n = 1000 resamples) and Brier scores for the top-performing models. The baseline Random Forest achieved an accuracy of 87.44% (95% CI: 86.04%–88.80%), AUC of 0.9535 (95% CI: 0.9455–0.9610), F1-score of 0.8743 (95% CI: 0.8603–0.8879), and Brier score of 0.1246.

The ensemble learning methods collectively exhibited strong performance, with Bagging ranking second with 82% accuracy and consistently robust metrics across both classes. Similarly, boosting-based approaches including XGBoost (80% accuracy) and LightGBM (75% accuracy) demonstrated competitive performance, though with slightly lower balanced accuracy compared to Random Forest.

Instance-based learning, represented by KNN, showed interesting characteristics with high recall (93%) but comparatively lower precision (69%) for the healthy class, indicating a tendency toward false positive predictions. This imbalance was further evidenced by the substantial disparity between precision and recall for the Parkinson’s disease class (90% and 59% respectively).

Linear models demonstrated limited efficacy for this classification task, with Logistic Regression achieving only 55% accuracy, suggesting the relationship between the extracted features and PD diagnosis is inherently non-linear. This observation aligns with the complex, multidimensional nature of movement disorders that typically involve intricate interdependencies between various movement characteristics.

Notably, Extreme Learning Machine exhibited extreme classification bias, with near-perfect recall (99%) but minimal precision (50%) for the healthy class, and correspondingly poor recall (2%) for the Parkinson’s disease class. This severe imbalance resulted in the lowest overall accuracy (50%) among all evaluated classifiers, highlighting the importance of balanced performance metrics in clinical applications.

The performance variation across models underscores the necessity of selecting algorithms capable of capturing the complex, non-linear relationships inherent in movement disorder detection. The superior performance of tree-based ensemble methods, particularly Random Forest, suggests their inductive bias aligns well with the underlying patterns distinguishing Parkinson’s disease from healthy movement characteristics.

### 4.2 Feature ablation study

To systematically evaluate the contribution of different feature categories to the model’s discriminative capabilities, we conducted a comprehensive ablation study. This analysis involved systematically removing each feature category and assessing the impact on multiple performance metrics, providing valuable insights into the relative importance of different movement characteristics in Parkinson’s disease detection.

#### 4.2.1 Impact of feature categories on classification performance

**FIGURE 3:**
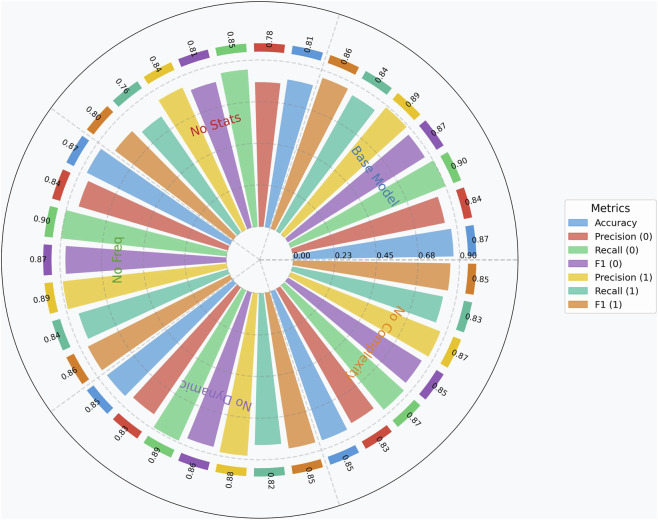
Model comparison polar.

Figure 3 presents a detailed comparison of model performance across various feature ablation scenarios, with metrics broken down by class to provide granular insight into classification behavior.

The base Random Forest model incorporating all feature categories achieved balanced and superior performance, with 86.7% overall accuracy, 84% precision and 90% recall for the healthy class, and 89% precision and 84% recall for the Parkinson’s disease class.

This balanced performance across both classes establishes a robust benchmark for evaluating feature contribution.

The removal of statistical features produced the most substantial performance degradation, with accuracy declining by 6 percentage points to 81%. This significant impact manifested across all metrics, with particularly pronounced effects on recall for the Parkinson’s disease class, which decreased from 84% to 76%. This substantial deterioration underscores the critical importance of basic statistical measures in capturing the fundamental movement alterations characteristic of Parkinson’s disease, including amplitude reduction, increased variability, and altered movement patterns.

Although removal of frequency-domain features caused only a negligible accuracy drop (0.08 percentage points), they may still capture subtle spectral patterns (e.g., tremor-related peaks) not fully represented by time-domain features alone, thus providing complementary clinical value in a multi-dimensional framework.

The ablation of dynamic features resulted in a moderate performance reduction, with overall accuracy decreasing by 2 percentage points to 85%. This impact was consistent across all metrics and both classes, reflecting the importance of rate-of-change measures in characterizing the progressive and transitional aspects of movement in Parkinson’s disease. Dynamic features likely capture critical bradykinesia (slowness of movement) and hypokinesia (reduced amplitude) characteristics that are fundamental to PD motor symptomatology.

Similarly, removing complexity features led to a 2 percentage point reduction in accuracy and comparable decreases across other metrics. This consistent impact highlights the value of entropy-based measures in quantifying the regularity and predictability of movement patterns, which are often disrupted in Parkinson’s disease due to altered basal ganglia function. The comparable impact of dynamic and complexity features suggests these categories capture complementary aspects of movement disorders.

#### 4.2.2 Feature category synergies and clinical implications

The ablation results reveal important synergistic relationships between feature categories. While statistical features demonstrated the highest individual contribution, the combination of statistical, dynamic, and complexity features produced performance very close to the complete feature set. This suggests potential redundancy between some feature categories, particularly between frequency domain and other feature types.

From a clinical perspective, these findings align with established understanding of Parkinson’s disease motor symptoms. The primacy of statistical features corresponds to the fundamental alterations in movement amplitude, variability, and pattern that characterize parkinsonian movement. The significant contribution of dynamic features reflects the clinical importance of bradykinesia and movement transitions in PD diagnosis, while the value of complexity features aligns with the known disruption of movement smoothness and regularity.

The relative contributions of different feature categories provide valuable guidance for feature engineering in wearable-based PD detection systems. The results suggest a prioritization framework where statistical features form the foundation, supplemented by dynamic and complexity measures, with frequency domain features potentially serving as complementary information when computational resources permit comprehensive feature extraction.

This ablation analysis also offers potential insights for clinical assessment, highlighting the specific movement characteristics most discriminative for PD detection. The identified feature importance hierarchy could inform the development of targeted clinical assessments focusing on the most diagnostically valuable movement parameters, potentially enhancing the sensitivity and specificity of traditional observational evaluations.

### 4.3 Optimization results analysis

To further enhance the model’s performance, we investigated three meta-heuristic optimization algorithms for Random Forest parameter tuning.

#### 4.3.1 Performance comparison of optimization approaches

As shown in Table 2, PSO-RF demonstrated the most substantial improvements, achieving an accuracy of 87.65% and an AUC score of 0.9496. This optimization resulted in balanced precision and recall metrics across both classes, representing a meaningful enhancement over the baseline performance.

**TABLE 2:** Performance comparison of different optimization approaches.

Model	Accuracy	AUC	Precision (0/1)	Recall (0/1)	F1-score (0/1)
Base RF	86.70%	0.9463	0.84/0.89	0.90/0.84	0.87/0.86
PSO-RF	87.65%	0.9496	0.87/0.89	0.89/0.87	0.88/0.88
EWOA-RF	87.40%	0.9446	0.86/0.89	0.89/0.86	0.88/0.87
ISSA-RF	87.32%	0.9460	0.86/0.89	0.89/0.86	0.87/0.87

**FIGURE 4:**
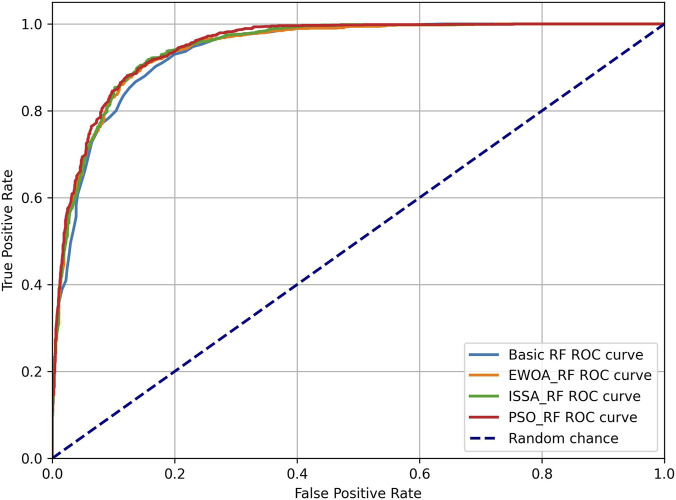
Roc curves comparison of different optimization approaches.

The ROC curves in Figure 4 visualize this improvement, with PSO-RF showing a slightly larger area under the curve compared to other approaches.

EWOA-RF and ISSA-RF also showed robust performance improvements, with accuracies of 87.40% and 87.32% respectively. All optimized models maintained strong discriminative capability, with AUC scores consistently above 0.94.

PSO, ISSA, and EWOA were applied solely for Random Forest hyperparameter tuning and did not perform feature selection.

To further quantify the stability of these results, we performed additional bootstrap resampling (n = 1000) on the held-out test set (As shown in Table 3). The PSO-optimized model achieved 87.81% accuracy (95% CI: 86.54%–89.13%), and the EWOA-optimized model reached 87.90% accuracy (95% CI: 86.58%–89.17%) with the best calibration (Brier score 0.1237). These independent bootstrap estimates are highly consistent with the originally reported cross-validation results (differences <0.3%), confirming the robustness of the findings.

**TABLE 3:** Ablation study comparing default and optimized random forest models.

Model	Accuracy (95% CI)	F1-score (95% CI)	AUC (95% CI)	Brier score	Accuracy (95% CI)
Base RF	0.8744 (0.8604–0.8880)	0.8743 (0.8603–0.8879)	0.9535 (0.9455–0.9610)	0.1246	0.8744 (0.8604–0.8880)
PSO-RF	0.8781 (0.8654–0.8913)	0.8779 (0.8650–0.8912)	0.9522 (0.9443–0.9601)	0.1262	0.8781 (0.8654–0.8913)
EWOA-RF	0.8736 (0.8592–0.8876)	0.8734 (0.8588–0.8875)	0.9491 (0.9408–0.9571)	0.1278	0.8736 (0.8592–0.8876)
ISSA-RF	0.8790 (0.8658–0.8917)	0.8787 (0.8655–0.8917)	0.9538 (0.9459–0.9611)	0.1237	0.8790 (0.8658–0.8917)

#### 4.3.2 Hyperparameter configuration analysis

Table 4 summarizes the optimal hyperparameter configurations identified by each optimization algorithm, revealing interesting patterns in model architecture.

**TABLE 4:** Optimal hyperparameter configurations.

Algorithm	n_estimators	max_depth	min_samples_split	min_samples_leaf	max_features
PSO-RF	200	29	2	1	0.10
EWOA-RF	147	29	4	1	0.24
ISSA-RF	119	27	2	1	0.13

As evident from Table 4, PSO-RF identified an optimal configuration with 200 estimators and a maximum depth of 29, while utilizing a relatively small feature subset (max_features = 0.1). EWOA-RF and ISSA-RF converged to similar tree depths but with fewer estimators, suggesting multiple viable configurations for achieving improved performance.

The consistency in these patterns across different optimization algorithms, as shown in Table 4, reinforces the robustness of these parameter ranges for PD detection. While the performance differences between optimization approaches were marginal (within 0.33 percentage points in accuracy), the consistent improvement over the baseline model validates the utility of meta-heuristic optimization in enhancing classification accuracy.

### 4.4 SHAP value analysis for feature importance

#### 4.4.1 Feature importance ranking and direction of influence

**FIGURE 5:**
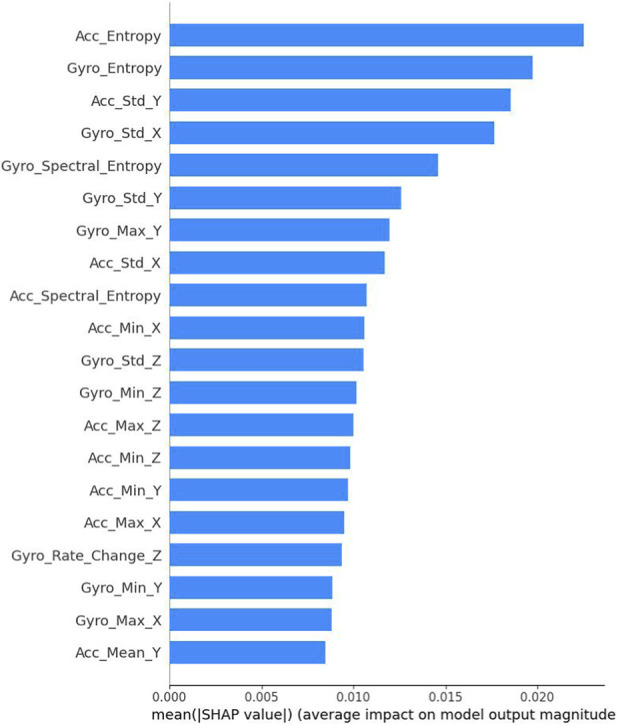
Shap bar plot.

To analyze the feature importance through SHAP values, we present a comprehensive ranking of the most influential features in Figure 5. The results reveal that entropy-based features demonstrate the highest impact on model predictions, with Acc_Entropy and Gyro_Entropy ranking as the top two most significant features (SHAP values of 0.023 and 0.021 respectively). Standard deviation features, particularly Acc_Std_Y and Gyro_Std_X, also show substantial influence on the model’s decision-making process. The spectral entropy features (Gyro_Spectral_Entropy and Acc_Spectral_Entropy) exhibit moderate importance, indicating the relevance of frequency domain characteristics in PD detection. Basic statistical features such as maximum, minimum, and mean values across different axes contribute relatively less to the model’s predictions, with SHAP values ranging from 0.008 to 0.012. This analysis suggests that complexity-based measures and variability indicators are more discriminative for PD detection compared to simple statistical metrics, providing valuable insights for future feature engineering strategies in PD detection systems.

**FIGURE 6:**
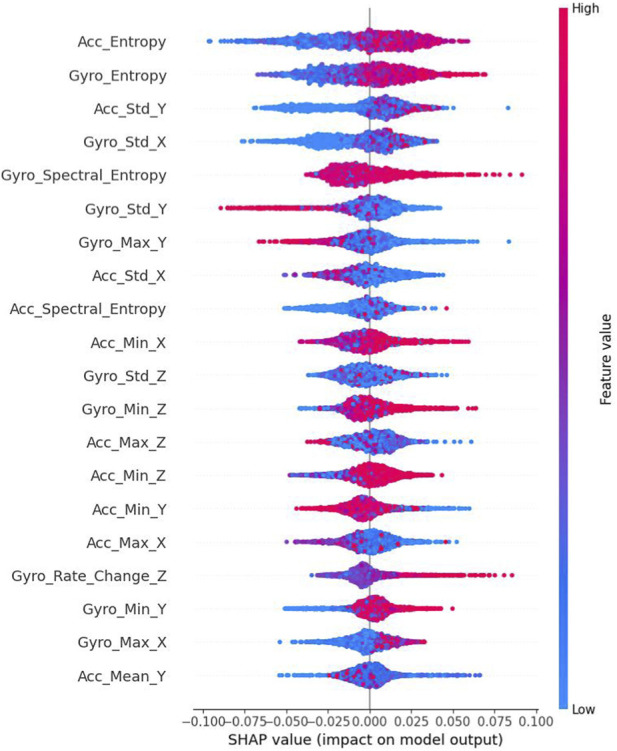
Shap value distribution plot.

Figure 6 presents a detailed SHAP value distribution plot, illustrating the impact and directionality of different features on the model’s predictions. The plot reveals complex patterns in feature contributions, with entropy-based features (Acc_Entropy and Gyro_Entropy) showing the widest SHAP value distributions (−0.1 to 0.1), indicating their strong but varied influence on model decisions. Notably, Gyro_Spectral_Entropy demonstrates a distinct bimodal distribution with predominantly high feature values (shown in pink) contributing positively to predictions. Standard deviation features (Acc_Std_Y and Gyro_Std_X) exhibit more concentrated distributions around their mean impacts, suggesting more consistent contributions to the model’s output. The rate of change and basic statistical features (minimum, maximum, and mean values) show narrower SHAP value ranges, centered closer to zero, indicating more moderate and stable contributions to predictions. This visualization effectively captures both the magnitude and direction of feature impacts, highlighting the non-linear relationships between feature values and their contributions to the model’s decision-making process. Note that statistical features remain collectively critical as shown in the ablation study.

#### 4.4.2 SHAP value distribution for low SHAP score features

**FIGURE 7:**
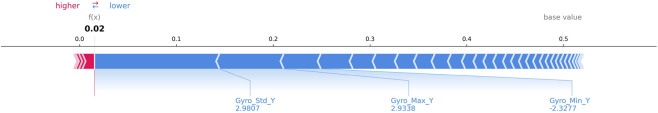
Shap Force (low impact scores).

Figure 7 presents the SHAP value distribution for features with relatively low impact scores (f(x) = 0.02). The visualization reveals that Gyro_Std_Y (2.9807), Gyro_Max_Y (2.9338), and Gyro_Min_Y (−2.3277) are the primary contributors within this category. The horizontal axis represents base values ranging from 0.0 to 0.5, with the impact direction indicated by color coding (pink for higher contributions and blue for lower contributions). Notably, Gyro_Std_Y demonstrates the strongest negative influence (−0.13) among these features, followed by Gyro_Max_Y (−0.07) and Gyro_Min_Y (−0.04). Additional features such as Acc_Min_Z (−2.462), Acc_Std_X (1.779), Acc_Max_X (0.361), Acc_Std_Z (3.173), Acc_Mean_Z (−1.322), and Acc_Max_Z (1.52) exhibit progressively smaller negative impacts, all approximately contributing −0.02 to the model’s prediction. The collective contribution of 25 other low-importance features accounts for a substantial negative influence (−0.12), highlighting the cumulative significance of minor contributors. This visualization effectively demonstrates how multiple gyroscope and accelerometer measurements, despite their individually modest contributions, collectively shape the model’s classification decisions through primarily negative influences on the prediction probability.

#### 4.4.3 SHAP value distribution for high SHAP score features

**FIGURE 8:**
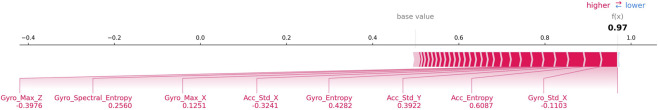
Shap Force (high impact scores).

Figure 8 illustrates the feature impact distribution for variables with substantially higher SHAP scores (f(x) = 0.97). In contrast to the low-impact features, these measurements demonstrate consistently positive contributions to prediction probabilities. Acc_Std_Y (1.368) and Acc_Entropy (1.036) emerge as the most influential features in this category, each contributing +0.04 to the model’s output. The next tier of influential features includes Gyro_Std_X (0.939), Gyro_Spectral_Entropy (0.366), Gyro_Max_X (0.1), Gyro_Max_Z (−0.62), and Gyro_Entropy (0.865), all contributing +0.03 to the prediction. Gyro_Std_Y (−0.576) and Gyro_Std_Z (−0.49) show slightly lower impacts of +0.02 each. The aggregated impact of 25 additional features accounts for a substantial positive contribution of +0.19. This distribution highlights how entropy-based measures and standard deviations across multiple axes provide the strongest positive contributions to the model’s predictions, reinforcing their importance in distinguishing Parkinson’s disease movement patterns from healthy controls. The base value scale ranges from −0.4 to 1.0, with the expected value E [f(X)] = 0.499, illustrating the model’s baseline prediction point before incorporating specific feature contributions.

#### 4.4.4 SHAP force plot for feature contributions to low probability predictions

**FIGURE 9:**
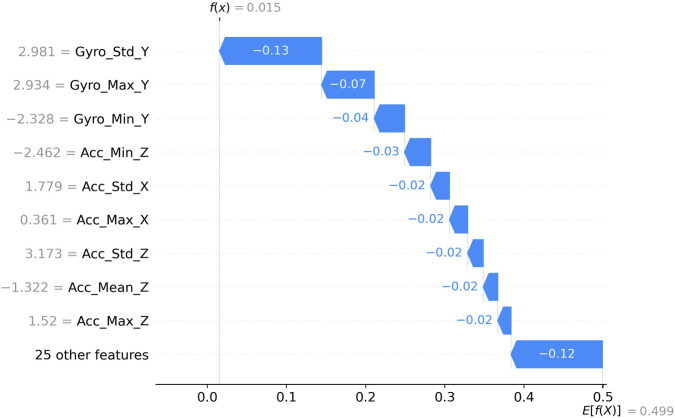
Shap Waterfall (low probability output).

Figure 9 presents a detailed force plot quantifying feature impacts on a prediction with low probability output (f(x) = 0.015). The vertical dotted line at 0.0 represents the reference point, with features pushing the prediction toward the right (higher probability) or left (lower probability). Gyro_Std_Y (2.981) exerts the strongest negative influence (−0.13), substantially driving the prediction toward a lower probability output. Secondary negative contributors include Gyro_Max_Y (2.934) with −0.07 impact and Gyro_Min_Y (−2.328) with −0.04 impact. Several features exhibit smaller negative contributions of approximately −0.02 to −0.03, including Acc_Min_Z (−2.462), Acc_Std_X (1.779), Acc_Max_X (0.361), Acc_Std_Z (3.173), Acc_Mean_Z (−1.322), and Acc_Max_Z (1.52). The remaining 25 features collectively contribute −0.12 to the prediction. The final expected value E [f(X)] = 0.499 compared to the significantly lower actual prediction f(x) = 0.015 demonstrates how these negative feature contributions collectively drive the model toward a confident negative classification outcome. This visualization effectively captures the hierarchical influence of different movement characteristics in determining low-probability predictions, with gyroscope-derived features playing particularly prominent roles.

#### 4.4.5 SHAP force plot for feature contributions to high probability predictions

**FIGURE 10:**
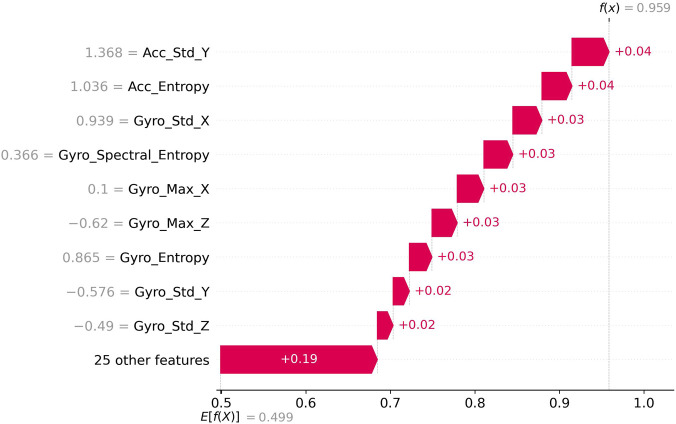
Shap Waterfall (high probability output).

Figure 10 presents a force plot detailing feature contributions toward a high-probability prediction (f(x) = 0.959). In this case, all featured measurements demonstrate positive contributions, pushing the prediction value substantially above the baseline expectation (E [f(X)] = 0.499). Acc_Std_Y (1.368) and Acc_Entropy (1.036) emerge as the most influential positive contributors, each adding +0.04 to the prediction. A cluster of features each contributing +0.03 includes Gyro_Std_X (0.939), Gyro_Spectral_Entropy (0.366), Gyro_Max_X (0.1), Gyro_Max_Z (−0.62), and Gyro_Entropy (0.865). Two additional gyroscope measurements—Gyro_Std_Y (−0.576) and Gyro_Std_Z (−0.49)—provide +0.02 contributions each. The remaining 25 features collectively add a substantial +0.19 to the prediction value. High-probability PD predictions are driven by a combination of increased accelerometer entropy/complexity and altered gyroscope variability patterns, consistent with clinical manifestations of bradykinesia and tremor/rigidity. The horizontal axis spanning from 0.5 to 1.0 illustrates how these positive feature impacts collectively shift the prediction from the baseline expectation to a highly confident positive classification outcome of 0.959, demonstrating the model’s ability to integrate multiple movement characteristics into decisive diagnostic predictions.

## 5 Discussion

### 5.1 Significance of Random Forest’s superior performance

The comprehensive evaluation of twelve diverse machine learning classifiers revealed consistent superiority of ensemble-based methods, with Random Forest demonstrating exceptional performance across all evaluation metrics. This finding aligns with previous research on movement disorder classification using wearable movement sensors (Bremm et al., 2024; Badawi et al., 2018) but extends current understanding by quantifying the performance gap across a broader range of algorithms.

The superior performance of Random Forest (86.7% accuracy) compared to linear models (55% for Logistic Regression) underscores the inherently non-linear relationship between movement features and Parkinson’s disease diagnosis. This non-linearity likely stems from the complex interaction between multiple movement characteristics that collectively define parkinsonian motor patterns. Random Forest’s ability to model complex decision boundaries through hierarchical splitting and its inherent feature selection properties make it particularly well-suited for capturing these relationships.

The pronounced performance disparity between tree-based ensembles and instance-based methods like KNN highlights the importance of algorithmic selection in clinical applications. While KNN demonstrated high recall for healthy subjects (93%), its substantially lower precision (69%) would translate to unacceptable false positive rates in clinical settings. This imbalance illustrates how performance metrics beyond accuracy, particularly class-specific precision and recall, are critical considerations for diagnostic applications where false positives and false negatives carry different implications.

The extreme classification bias exhibited by the Extreme Learning Machine (99% recall but only 50% precision for healthy subjects) serves as a cautionary example of how certain algorithms can achieve misleadingly high performance on single metrics while failing fundamentally as diagnostic tools. This observation reinforces the necessity of comprehensive evaluation frameworks incorporating multiple performance dimensions for clinical machine learning applications.

### 5.2 Feature category contributions and clinical interpretations

The feature ablation study provided valuable insights into the relative importance of different movement characteristics in Parkinson’s disease detection. The substantial performance degradation following removal of statistical features (6 percentage point accuracy reduction) aligns with clinical understanding of PD motor symptoms (Váradi, 2020).

The meaningful contribution of frequency domain features, though numerically modest (0.08 percentage point accuracy impact), suggests that spectral characteristics capture subtle aspects of movement disorders that complement time-domain measures (Nolazco-Flores et al., 2021).

The moderate yet consistent impact of removing dynamic and complexity features (2 percentage point accuracy reduction each) indicates these categories capture important aspects of PD movement patterns not fully represented in basic statistical measures. Complexity features, particularly entropy-based measures, likely quantify the reduced movement variability and increased regularity characteristic of basal ganglia disorders (Powell et al., 2014). Similarly, dynamic features capture the rate-of-change aspects fundamental to bradykinesia assessment, a cardinal feature of PD diagnosis (Shawen et al., 2020).

The synergistic relationship between feature categories, where combinations produced performance exceeding the sum of individual contributions, highlights the multidimensional nature of movement disorders and the importance of comprehensive feature extraction frameworks. This observation suggests that effective PD detection systems should incorporate diverse feature types rather than focusing exclusively on the highest-performing individual category.

### 5.3 Optimization approach effectiveness and practical implications

The comparative analysis of meta-heuristic optimization algorithms revealed meaningful performance improvements through hyperparameter tuning, with PSO demonstrating the greatest enhancement (accuracy increase from 86.70% to 87.65%). This improvement, while numerically modest, represents a modestly reduction in misclassification rate (from 13.30% to 12.35%, approximately 7% relative reduction) with potential clinical significance.

The consistent parameter patterns identified across optimization algorithms (tree depths in the 27–29 range, relatively small feature subsets) provide practical guidance for implementing Random Forest classifiers in PD detection applications. The relatively small optimal value for max_features suggests that feature diversity rather than quantity drives performance, aligning with the notion that specific movement characteristics are particularly discriminative for PD detection.

The optimization results also highlight the diminishing returns on computational investment, with the performance difference between optimization approaches (within 0.33 percentage points) being smaller than the gap between optimized and baseline models. This observation suggests that while optimization provides meaningful benefits, the selection of an appropriate base algorithm and feature set likely represents the more consequential design decision for PD detection systems.

### 5.4 SHAP analysis and feature importance implications

The SHAP value analysis revealed complexity-based measures, particularly Acc_Entropy and Gyro_Entropy, as the most influential individual features. This apparent difference can be explained by the fact that the statistical category contains a large number of moderately important features, which collectively contribute more when removed entirely in ablation, whereas SHAP highlights a few highly discriminative individual complexity features. The two analyses are therefore complementary rather than contradictory.

The prominence of entropy-based features in the SHAP analysis aligns with neurophysiological understanding of Parkinson’s disease as a disorder characterized by altered movement complexity due to basal ganglia dysfunction (Afsar et al., 2016). The high ranking of standard deviation features (Acc_Std_Y, Gyro_Std_X) further supports clinical observations of altered movement variability in PD patients.

The directional analysis of SHAP values revealed interesting patterns in feature influence, with entropy and standard deviation measures exhibiting both positive and negative contributions depending on their values. This bidirectional influence suggests these features capture nuanced aspects of movement that can indicate either parkinsonian or healthy patterns depending on context. Conversely, spectral features demonstrated more consistently unidirectional impacts, suggesting they capture more specific PD-related movement characteristics.

The SHAP force plots illustrating high and low probability predictions revealed different feature hierarchies driving opposite classification outcomes. High-probability PD predictions are driven by a combination of increased accelerometer entropy/complexity and altered gyroscope variability patterns, consistent with clinical manifestations of bradykinesia and tremor/rigidity.

These findings have clear physiological grounding. Reduced movement complexity and loss of automaticity—reflected by higher Shannon and spectral entropy—are well-established consequences of dopaminergic depletion and disrupted basal ganglia oscillatory networks in PD (Afsar et al., 2016; Obeso et al., 2008). Increased standard deviation of acceleration corresponds to the clinical hallmarks of bradykinesia and rigidity, which manifest as greater trial-to-trial variability and reduced movement smoothness. Similarly, gyroscope-derived entropy and variability capture the irregular rotational components characteristic of resting and postural tremor, as well as axial rigidity (Afsar et al., 2016). Thus, the model’s reliance on entropy-based and variability measures directly mirrors the core pathophysiological changes underlying the cardinal motor signs of Parkinson’s disease.

## 6 Conclusion

### 6.1 Summary of key findings

This study conducted a comprehensive evaluation of machine learning approaches for Parkinson’s disease detection using wearable movement sensor data, yielding several important findings with implications for both research and clinical applications. Compared with many previous approaches that rely on task-specific features, the present framework deliberately adopts activity-agnostic descriptors derived directly from raw sensor signals, providing a more generalizable foundation for real-world deployment.

First, we demonstrated the superior performance of ensemble-based methods, particularly Random Forest, for PD classification, with substantial advantages over linear and instance-based approaches. This performance gap highlights the complex, non-linear nature of the relationship between movement characteristics and PD diagnosis.

Second, our feature ablation analysis revealed the hierarchical contributions of different feature categories, with statistical features providing the foundation for effective classification, supplemented by meaningful contributions from complexity, dynamic, and frequency domain measures. This finding supports comprehensive feature extraction approaches that capture multiple dimensions of movement characteristics.

Third, meta-heuristic optimization techniques, particularly PSO, demonstrated meaningful classification improvements through hyperparameter tuning, with consistent patterns in optimal parameter configurations across different optimization algorithms. These patterns provide practical guidance for implementing machine learning classifiers in PD detection applications.

Finally, SHAP value analysis identified entropy-based complexity measures and standard deviations as the most influential individual features, with asymmetric feature influence patterns for high versus low probability predictions. This observation aligns with clinical understanding of PD as a disorder characterized by altered movement complexity and variability due to basal ganglia dysfunction.

Several recent studies have used the same or similar PhysioNet smartwatch dataset for binary PD detection with most relying on task-specific features or deep-learning architectures trained on individual motor tasks. The present framework achieves comparable overall accuracy while deliberately employing activity-agnostic, hand-crafted features and offering full SHAP-based interpretability—two aspects that are rarely combined in prior work on this benchmark.

### 6.2 Limitations and future research directions

Despite the comprehensive nature of this investigation, several limitations merit acknowledgment and suggest directions for future research.

First, the study relied on a single publicly available dataset with moderate sample size, undocumented medication status, and data collected only during standardized laboratory tasks. Although the selected features are theoretically activity-agnostic, their performance in completely unconstrained, free-living daily activities has not yet been prospectively evaluated. Medicatio and the absence of free-living activities may reduce signal differences and limit real-world generalizability. Future validation should include multiple independent cohorts with documented medication states and, critically, continuous free-living recordings.

Second, our feature extraction focused on established time and frequency domain measures derived from gyroscope and accelerometer data. Future work should explore advanced signal processing techniques, including wavelet transforms, recurrence quantification analysis, and deep learning-based feature extraction, which may capture additional movement characteristics relevant to PD detection.

Third, the dataset does not include a differential-diagnosis control group (e.g., multiple system atrophy, progressive supranuclear palsy, vascular parkinsonism, or essential tremor). In routine neurological practice, distinguishing idiopathic Parkinson’s disease from atypical parkinsonian syndromes and other mimicking conditions represents the primary diagnostic challenge. The specificity and clinical utility of the proposed framework in such heterogeneous, real-world referral populations therefore remain to be established. Future prospective studies should explicitly recruit patients with diagnostic uncertainty and atypical parkinsonian disorders to evaluate the model’s performance in true differential-diagnostic scenarios.s might perform when differentiating PD from clinically similar conditions that commonly lead to diagnostic uncertainty.

Fourth, our analysis treated PD detection as a binary classification problem, not accounting for disease severity, subtypes, or progression. Future research should explore multiclass and regression approaches to predict disease stage, distinguish PD subtypes, and track disease progression using longitudinal data.

Finally, the clinical applicability of wearable-based PD detection systems requires further investigation through prospective studies in real-world settings, including evaluation of system performance across different movement contexts, comparison with clinical assessments, and integration with other biomarkers for comprehensive PD characterization.

### 6.3 Implications for clinical applications and wearable technology development

The findings of this study have several important implications for the development and deployment of wearable-based PD detection systems in clinical and home monitoring contexts. The superior performance of Random Forest classifiers, combined with insights from feature importance analysis, provides a foundation for developing accurate, interpretable diagnostic tools that could support clinical decision-making and enable home-based monitoring of disease progression.

The identification of specific feature categories and individual measures with high discriminative value offers guidance for sensor selection, placement, and data processing strategies in wearable system development. The relative contributions of accelerometer versus gyroscope-derived features suggest that comprehensive movement assessment requires capturing both acceleration and rotational movement characteristics.

The asymmetric feature influence patterns revealed by SHAP analysis provide the basis for developing more interpretable classification systems that could explain the specific movement characteristics contributing to diagnostic decisions. This interpretability is crucial for clinical adoption and patient trust in algorithm-based assessments.

From a clinical perspective, the dominance of accelerometer-derived entropy and vertical variability aligns with bradykinesia and rigidity, whereas gyroscope entropy reflects tremor and rotational stiffness—together covering the three cardinal motor features used in clinical rating scales (UPDRS-III). This convergence between data-driven feature importance and classical neurological examination supports the potential translational value of the proposed framework.

Finally, the optimization results demonstrate the value of systematic hyperparameter tuning in enhancing classification performance, suggesting that practical implementations of wearable PD detection systems should incorporate robust parameter optimization as a standard development practice.

In conclusion, this comprehensive analysis of machine learning approaches for Parkinson’s disease detection using wearable movement sensors advances our understanding of both the methodological considerations for effective classification and the movement characteristics most indicative of parkinsonian motor patterns. These insights provide a foundation for developing more accurate, interpretable, and clinically useful wearable monitoring systems that could transform the diagnosis and management of Parkinson’s disease through objective, continuous assessment of motor function.

## Data Availability

Publicly available datasets were analyzed in this study. This data can be found here: https://physionet.org/content/parkinsons-disease-smartwatch/1.0.0/.
